# Identification of a novel Polo-like kinase 1 inhibitor that specifically blocks the functions of Polo-Box domain

**DOI:** 10.18632/oncotarget.13603

**Published:** 2016-11-25

**Authors:** Yunyu Chen, Jing Zhang, Dongsheng Li, Jiandong Jiang, Yanchang Wang, Shuyi Si

**Affiliations:** ^1^ Institute of Medicinal Biotechnology, Peking Union Medical College & Chinese Academy of Medical Sciences, Beijing 100050, China; ^2^ Department of Biomedical Sciences, College of Medicine, Florida State University, Tallahassee, FL 32306, USA

**Keywords:** Polo-like kinase 1 inhibitor, Polo-Box domain, fluorescence polarization, protein-protein interactions, cancer therapy

## Abstract

Polo-like kinase 1 (Plk1) is a promising target for cancer therapy due to its essential role in cell division. In addition to a highly conserved kinase domain, Plk1 also contains a Polo-Box domain (PBD), which is essential for Plk1's subcellular localization and mitotic functions. We adopted a fluorescence polarization assay and identified a new Plk1 PBD inhibitor T521 from a small-molecule compound library. T521 specifically inhibits the PBD of Plk1, but not those of Plk2-3. T521 exhibits covalent binding to some lysine residues of Plk1 PBD, which causes significant changes in the secondary structure of Plk1 PBD. Using a cell-based assay, we showed that T521 impedes the interaction between Plk1 and Bub1, a mitotic checkpoint protein. Moreover, HeLa cells treated with T521 exhibited dramatic mitotic defects. Importantly, T521 suppresses the growth of A549 cells in xenograft nude mice. Taken together, we have identified a novel Plk1 inhibitor that specifically disrupts the functions of Plk1 PBD and shows anticancer activity.

## INTRODUCTION

Polo-like kinases (Plks) are a conserved family of serine/threonine kinases, which are essential for multiple steps in mitosis [1]. Among the five identified mammalian Plk family members, Plk1-3 show more sequence similarity [2]. Plk1-3 have a unique C-terminal noncatalytic region containing two tandem Polo boxes and a highly conserved N-terminal catalytic kinase domain [3]. The Polo-box domain (PBD) of Plk1 is crucial for its subcellular localization and mitotic functions, because the PBD binds to phosphorylated substrates that contain a consensus S-pS/pT-P/X motif [4, 5].

Plk1 regulates several critical mitotic events, such as mitotic entry, centrosome maturation, spindle assembly, chromosome segregation and cytokinesis [1, 6, 7]. Because overexpression of Plk1 has been reported in many human cancers, it is regarded as a potential prognostic marker for cancer patients [8–10]. Inhibition of Plk1 activity causes mitotic arrest and apoptotic cell death in most cancer cell lines. Plk1 inhibitors reduce tumor proliferation, but have little effect on normal cells based on the results from mouse xenograft models [11]. Hence, Plk1 is believed to be an attractive therapeutic target for cancer cells.

Unlike Plk1 that promotes cell cycle progression, Plk2 and Plk3 have been identified as tumor suppressors to prevent mitotic catastrophe and preserve genomic integrity [7, 12]. Significantly lower expression of Plk2 has been observed in a wide range of B-cell neoplasms due to CpG methylation that causes transcriptional silencing of Plk2, supporting the role of Plk2 as a tumor suppressor in hematopoietic diseases [13]. The function of Plk4 is not fully understood yet, but evidence suggests that it may ensure genome stability by promoting centriole duplication [14, 15]. Plk5 is highly expressed in the central nervous system and has been reported as a glioblastoma suppresser [16]. Therefore, specific inhibition of Plk1 kinase activity, but not other Plks, could be important for cancer therapy.

Many Plk1 inhibitors targeting the kinase domain have been identified and some are currently tested in clinical trials for cancer treatment, such as BI6727 (volasertib), ON01910 (rigosertib), BI2536, GSK461364, and HMN-214 [17, 18]. Recently, volasertib has been evaluated in Phase III clinical trials and shows promising clinical efficacy against acute myeloid leukemia (AML), for which it received a breakthrough therapy designation from the US FDA in 2013. Moreover, a phase II trial for volasertib in the treatment of advanced non-small cell lung cancer (NCT00824408) is underway [18, 19]. Rigosertib is now in phase III trials for the second-line treatment of high-risk myelodysplastic syndrome (NCT01928537 and NCT00906334) and phase II trials for the first-line treatment of low-risk myelodysplastic syndrome (NCT01584531 and NCT01904682) [20]. A phase III study of rigosertib in combination with gemcitabine is also underway for patients with metastatic pancreatic cancer (NCT01360853) [18, 21]. The ATP-binding pocket of kinases is a classical target for kinase inhibitors, but these inhibitors frequently face drug resistance because of mutations in the ATP-binding pocket [22]. Another disadvantage of these inhibitors is the lack of the specificity due to the conserved nature of ATP-binding pocket of all kinases.

The unique PBD of Plk1 is a promising target for Plk1 inhibitors, because the inhibition could be more specific and show less drug resistance. Poloxin [23, 24], Thymoquinone (TQ) [23], Poloxipan [25], Purpurogallin (PPG) [26] and Green Tea Catechins [27] have recently been identified as Plk1 inhibitors specific for the PBD. However, these compounds inhibit both Plk1 and Plk2, although they show less inhibitory effect on Plk3 [23, 25–27]. Given the limited number of Plk1 PBD inhibitors and their modest selectivity, we performed high-throughput screen for small molecules that specifically inhibit Plk1 PBD. As a result, we identified a novel Plk1 PBD inhibitor with distinct chemical scaffold that exhibits stronger specificity toward Plk1 than other inhibitors.

## RESULTS

### Establishment of fluorescence polarization-based high-throughput screening system for Plk1 PBD inhibitors

**Figure 1:**
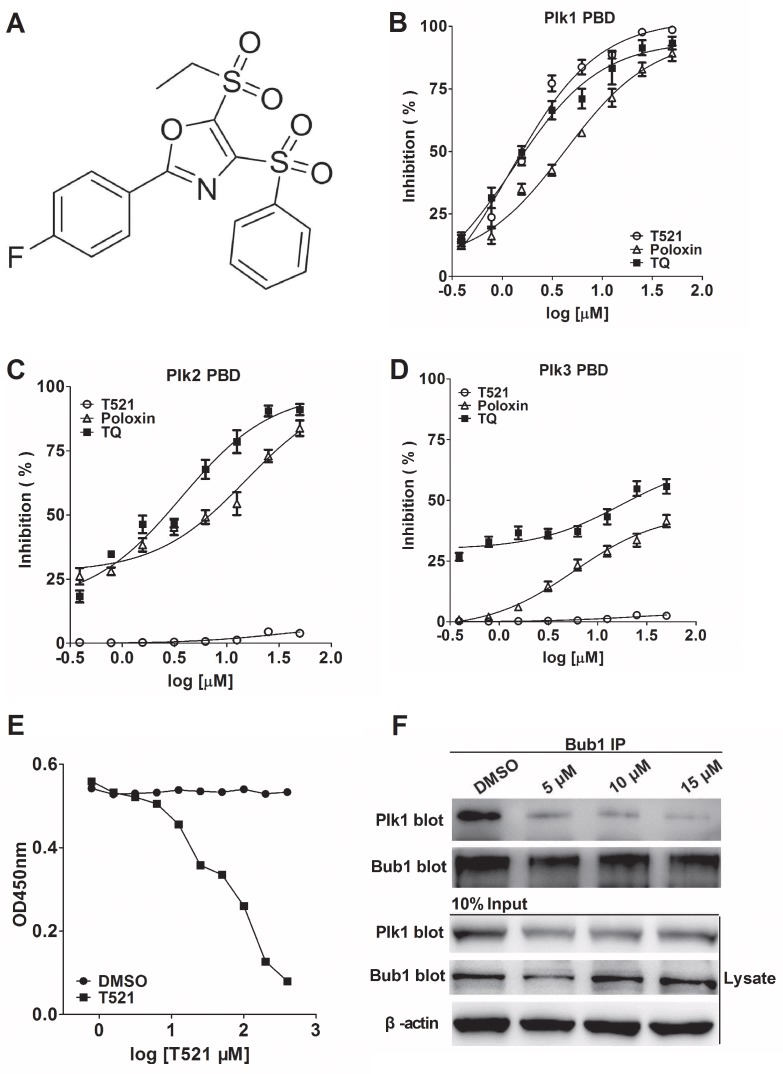
Identification of a potent small molecule inhibitor of Plk1 PBD **A.** The chemical structure of T521: 5-(ethylsulfonyl)-2-(4-fluorophenyl)-4-(phenylsulfonyl) oxazole. **B, C, D.** The inhibitory effect of T521, Poloxin and TQ on the binding of the PBDs of Plk1, 2 and 3 to the FITC-labeled phosphopeptide was analyzed by FP assay. Briefly, T521, Poloxin or TQ was incubated with Plk1-3 PBD for 1 hr at room temperature (RT) prior to addition of FITC-labeled phosphopeptide. The inhibitory effect was calculated as described in the Materials and Methods. Error bars represent SD. **E.** ELISA-based PBD binding inhibition assay to determine the inhibitory effect of T521 on the interaction between Plk1 PBD and its binding target Map205^PBM^. His-tagged PBD of Plk1 and different concentrations of T521 were added into GST-Map205^PBM^-coated plates. After incubation, the plate was washed and then probed with mouse anti-His primary antibody, followed by goat anti-mouse IgG-HRP secondary antibody. After washing, the plate was incubated with TMB solution and the OD_450_ was measured. Detailed procedure was described in Methods and Materials. DMSO was used as control. **F.** T521 inhibits Bub1-Plk1 interaction *in vivo* using Co-immunoprecipitation (IP) assay. HeLa cells synchronized by double-thymidine block were released into medium containing DMSO or T521 at indicated concentrations for 10 hrs, and then lysed and subject to Bub1 IP. The proteins in the precipitates were separated by 10% SDS-PAGE and probed with the anti-hPlk1 and anti-Bub1 antibodies. Moreover, the Bub1 and Plk1 levels were also determined in the lysates of HeLa cells before IP. β-actin served as the loading control.

Fluorescence polarization (FP) allows rapid and quantitative analysis of diverse molecular interactions and enzyme activities, and it was adopted for high-throughput screening for drug discovery in the mid-1990s [28]. Based on the consensus PBD binding motif S-pS/pT-P/X [5], we have adopted a FP screening system to identify small molecules that specifically bind to Plk1 PBD and disrupt PBD-substrate interactions. The FP screening assay was established as described previously [29, 30]. In our assay, the FITC-Poloboxtide was chemically synthesized and served as Plk1 PBD aptamer. Because FP assay monitors the increase in rotational mobility of a relatively small molecule upon separation from a large binding partner, the compounds that disrupt the binding between Plk1 PBD and FITC-Poloboxtide would lower the value of mP (millipolarization units). From a random compound library purchased from ChemDiv (20,000 compounds), T521 was identified as a potent Plk1 PBD inhibitor with an IC_50_ of 1.22±0.13 μM, while Poloxin and TQ displayed an IC_50_ of 4.27±0.69 μM and 1.36±0.38 μM, respectively (Figure 1A, 1B, Table 1). Hence, T521 and TQ exhibit similar inhibitory effect against Plk1 PBD, but Poloxin is relatively less effective. Interestingly, the FP assay indicates that T521 shows little inhibition for the binding of the PBDs of Plk2-3 to FITC-labeled phosphopeptide at a concentration as high as 500 μM (Figure 1C, 1D, Table 1). Therefore, compared with the identified Plk1 PBD inhibitors Poloxin and TQ, T521 exhibits much more selectivity against Plk1 PBD *in vitro*.

**Table 1:** The selectivity of T521, Poloxin and TQ against the PBDs of Plk1-3 *in vitro*

Plks PBD	T521 Apparent IC_50_	Poloxin Apparent IC_50_	TQ Apparent IC_50_
Plk1 PBD	1.22±0.13 μM	4.27±0.69 μM	1.36±0.38 μM
Plk2 PBD	>500 μM	15.62±2.26 μM	3.65±0.96 μM
Plk3 PBD	>500 μM	46.68±3.55 μM	18.86±1.32 μM

Map205 (microtubule-associated protein 205) localizes at the mitotic apparatus and cytoplasmic microtubules [31]. It binds to the PBD of human Plk1 with strong affinity and specificity, but the binding is independent of phosphorylation [32, 33]. Moreover, the crystal structure confirms the binding of zebrafish Plk1 PBD with Map205^PBM^ (PBD-binding motif of *Drosophila* Map205), and the binding site of Map205 on the Plk1 PBD largely overlaps with the phosphopeptide-binding site. Furthermore, Map205^PBM^ could efficiently block the interaction between Plk1 PBD and FITC-Poloboxtide using FP assay [34]. Inspired by these reports, we examined the effect of T521 on the interaction between Plk1 PBD and GST-Map205^PBM^ (276-325 aa) by a competitive ELISA. As shown in Figure 1E, T521 inhibited the binding between Plk1 PBD and GST-Map205^PBM^, but the IC_50_ is relatively high (IC_50_ of 90 μM), which could be attributed to the strong Map205^PBM^-PBD interaction [33]. Taken together, these results indicate that T521 is able to perturb the interaction of Plk1 PBD with its substrates *in vitro* and displays more desirable specificity toward Plk1 than Poloxin and TQ [23].

Budding uninhibited by benzimidazole 1 (Bub1), a spindle assembly checkpoint protein, is required for the kinetochore localization of Plk1 in mitosis through a direct interaction between Bub1 and the PBD of Plk1 [35]. To analyze if T521 blocks Plk1-Bub1 interaction *in vivo*, we used anti-Bub1 antibody to immunoprecipitate Bub1 from HeLa cells released from double-thymidine arrest into medium containing T521 (5, 10, 15 μM) for 10 hrs. The protein levels of Bub1 and Plk1 in the inputs and the immunoprecipitates were detected, respectively. A clear decrease of Plk1 protein level in the immunoprecipitates was observed in cells treated with T521, indicating that T521 disrupts Plk1-Bub1 interaction *in vivo* (Figure 1F). We noticed the uneven protein levels of Plk1 and Bub1 in the inputs, which could be a result of different cell cycle stages caused by T521 treatment. Because Bub1 binds to the Plk1 PBD, this result supports the notion that T521 disturbs the functions of Plk1 PBD *in vivo.*

### The binding mode of T521 to Plk1 PBD

**Figure 2:**
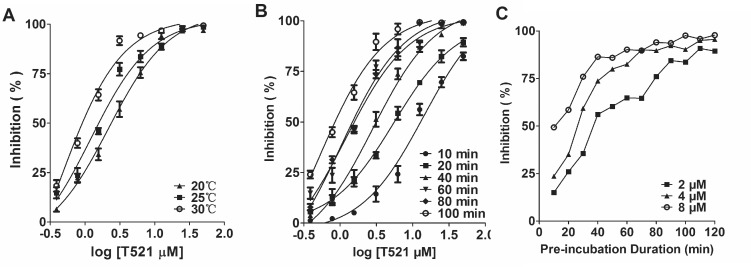
The binding mode of T521 to Plk1 PBD **A.** The inhibition of Plk1 PBD by T521 at different temperatures. Certain concentrations of T521 were incubated with Plk1 PBD for 1 hr at indicated temperatures, and then FITC-Poloboxtide was added into the mixture. FP assay was performed after 15 min incubation. Here shows the average of three independent experiments. Error bars represent SD. **B.** Time-dependent inhibition of Plk1 PBD by T521. Certain concentrations of T521 were incubated with Plk1 PBD for indicated times at RT, and then FITC-Poloboxtide was added into the mixture. FP assay was performed after 15 min incubation. Here shows the average of three independent experiments and error bars represent SD. **C.** Indirect analysis of the T521/Plk1 PBD binding kinetics using the FP assay. Plk1 PBD was incubated with T521 (2, 4, and 8 μM) for the indicated times and then FITC-Poloboxtide was added to the mixture for further incubation (15 min). Then FP assay was performed to analyze the binding between PBD and FITC-Poloboxtide, which indirectly reflects the occupancy of PBD by T521. Detailed protocol was described in Methods and Materials.

The covalent binding mode was speculated from the interactions between Poloxin, TQ and Plk1 PBD [23]. We found that the inhibition of the interaction between Plk1 PBD and FITC-Poloboxtide by T521 was more efficient at higher temperatures (Figure 2A), and the inhibition increased over time (Figure 2B), indicating a covalent binding. To test this possibility, we incubated purified Plk1 (10 μM) with T521 (500 μM) in 50 mM Tris-HCl (pH8.0) at room temperature for 30 min. HPLC-Q-TOF mass spectrometry analysis was performed and the intact mass of Plk1 protein increased by 907.05 Da (Supplementary Figure S1A and S1B), suggesting covalent addition of three T521 fragments (MW=301 Da) to Plk1. In contrast, no addition of T521 to Plk2 or Plk3 was detected after their incubation with T521 (Supplementary Figure S1C, S1D, S1E and S1F). The subsequent protease digestion and Nano-LC-LTQ-Orbitrap XL MS analysis demonstrated that the covalent binding sites of Plk1 are K209, K388 and K574. Two of them (K388 and K574) reside within the Plk1 PBD, however these binding sites do not locate within the classic phosphopeptide binding pocket (Supplementary Figure S2).

Circular dichroism (CD) has been widely used to examine protein structures in solution. In the far UV spectral region (180-240nm), which corresponds to peptide bond absorption, the CD spectrum can give some information about the secondary structural features of a protein, such as α-helix and β-sheet [36]. The CD spectra of Plk1 PBD show significant secondary structure changes after T521 binding (Supplementary Figure S3). The difference between the complex of Plk1 PBD with T521 and native Plk1 PBD is reflected by the composition of α-helix (11.63% vs 26.18%), β-turn (29.59% vs 21.08%), and random coil (41.46% vs 34.80%). Because the native conformational structure of PBD is necessary for phosphopeptide binding [5], these secondary structure changes caused by T521 covalent binding may contribute to its strong inhibition of PBD functions.

We further performed a FP assay to characterize the binding affinity between T521 and Plk1 PBD. Plk1 PBD was first incubated with T521 for different times, and then FITC-Poloboxtide was added into the mixture. After 15 min, the fluorescence signal was measured. We noticed the presence of more unbound FITC-Poloboxtide as the pre-incubation time or T521 concentration increases, indicating that the presence of Poloboxtide fails to disrupt T521-Plk1 PBD interaction. However, different concentrations of T521 showed the similar inhibitory effect when pre-incubation was long enough (Figure 2C). We speculate that the T521-Plk1 PBD binding kinetics is slow, but the complex is stable once formed. These results are also consistent with the covalent-binding mode between T521 and Plk1 PBD.

### The effect of T521 on the proliferation of various cell lines

We next determined the effect of T521 on the proliferation of cancer and normal cell lines. T521 efficiently inhibited the proliferation of all tested 12 human cancer cell lines with IC_50_ ranging from 1 to 5 μM. Moreover, the proliferation of three exponentially growing normal cell lines, MRC5, HEK-293 and L02 cells, was also blocked by T521 at IC_50_ ranking from 3 to 10 μM, indicating the cytotoxicity of T521 (Table 2). We did not find obvious difference between tumor and normal cell lines in response to T521, at least in the cell culture system. This observation is consistent with the known Plk1 inhibitors Poloxin and BI2536 that inhibit the proliferation of cancer cells as well as normal cells *in vitro* [24, 37].

**Table 2:** The inhibitory effect of T521 on the proliferation of various cell lines

Cell lines	Tissue type	IC_50_(μM)
Tumor		
MCF-7	Breast	1.58
HepG2	Liver	3.17
Bel7402	Liver	5.13
MG63	Bone	6.25
SaOS-2	Bone	1.56
U-2OS	Bone	1.06
SH-SY5Y	Brain	1.37
HeLa	Cervix	4.43
HCT-116	Colon	3.55
HT29	Colon	3.86
PC3	Prostate	1.09
A549	Lung	3.56
Normal		
MRC-5	Lung	2.64
HEK-293	Kidney	8.66
L02	Liver	7.11

### The Effect of T521 on cell cycle progression of HeLa cells

**Figure 3:**
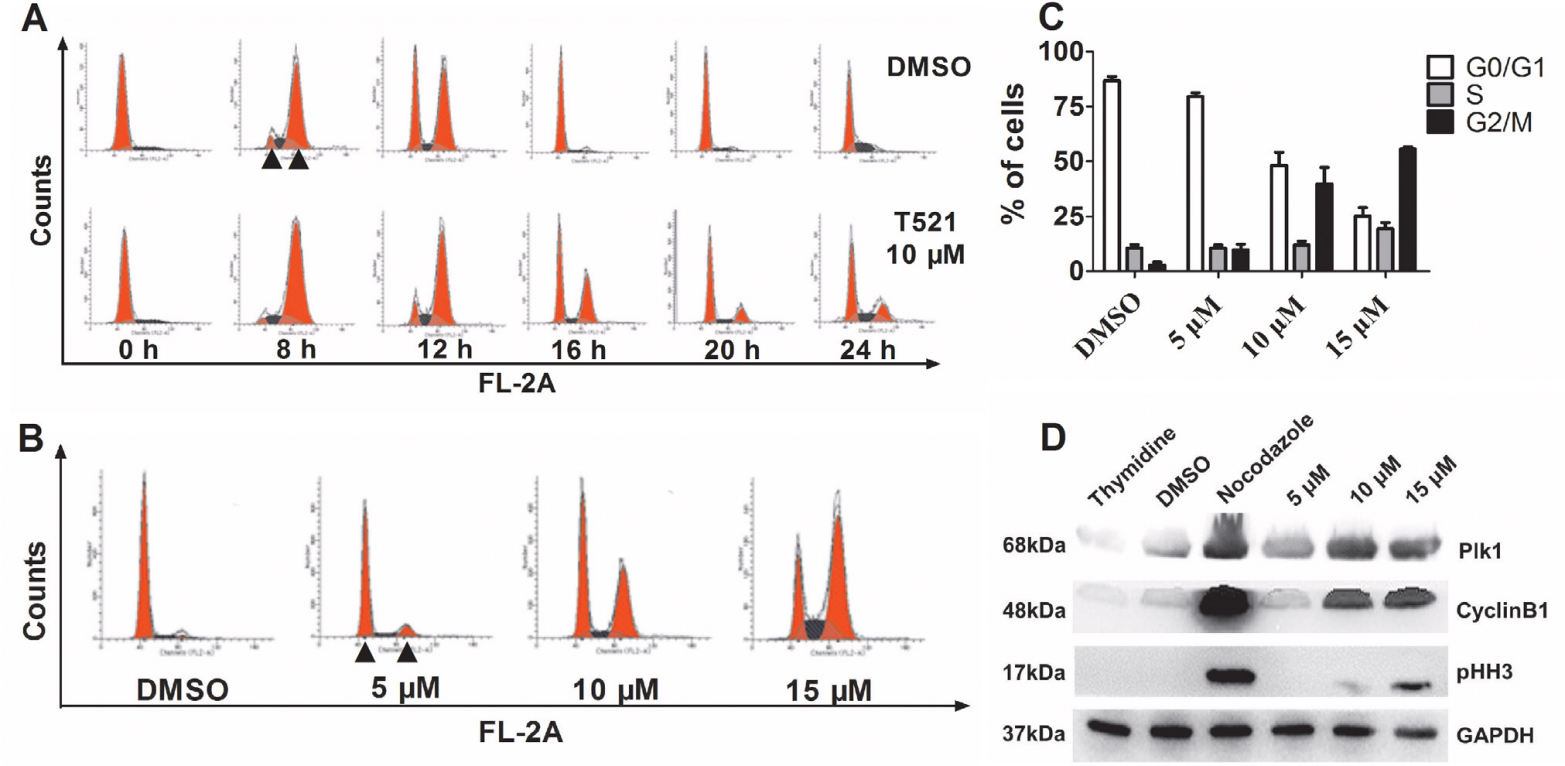
T521 induces mitotic arrest in HeLa cells **A.** HeLa cells (1.0×10^5^ cell/well) were synchronized at the G_1_/S boundary by double-thymidine block, and then released into medium with or without T521 (10 μM). Cell cycle progression was analyzed for DNA content by flow cytometry. DNA contents of 2C (left) and 4C (right) are indicated by triangles in the image. **B.** The G_2_/M arrest induced by different concentrations of T521 after release from double-thymidine block for 16 hrs. Cell cycle analysis was performed as described in A. **C.** Quantitative analysis of cell cycle distribution of DMSO or T521-treated HeLa cells in panel B (n=3). Error bars represent SD. **D.** Protein levels of mitotic markers in T521-treated HeLa cells. Synchronized HeLa cells were released into medium containing T521 at the indicated concentrations or nocodazole (50 ng/mL) for additional 16 hrs. Cellular extracts were prepared and the protein levels of Plk1, Cyclin B1 and pHH-3 were analyzed by western blotting. Nocodazole-treated cells were used as a control for mitotic arrest. DMSO-treated cells and cells synchronized with thymidine (2 mM) were also used as controls. GAPDH was used as the loading control.

Previous studies have demonstrated the essential functions of Plk1 in G_2_/M transition [1, 11]. Next, we examined the effect of T521 on the cell cycle progression of HeLa cells synchronized by double-thymidine block. After release from the G_1_/S arrest, the DNA content was monitored over time using fluorescence-activated cell sorter analysis (Figure 3A). In the absence and presence of T521 (10 μM), the cells completed S-phase and entered the G_2_/M phase after release for 8 hrs as indicated by the increase of cell population with 4C content DNA. After release for 16 hrs, most control cells entered G_1_-phase with 2C DNA content, but about 40% of T521-treated HeLa cells still showed 4C DNA content. At least 20% of HeLa cells treated with T521 remained in G_2_/M phase during the entire course (24 hrs) of this experiment. Therefore, treatment with 10 μM T521 causes obvious cell cycle delay in G_2_/M phase, but cell cycle progression cannot be blocked completely at this concentration.

We further found that T521 induces more dramatic G_2_/M delay of HeLa cells at higher concentrations (Figure 3B and 3C). Consistent with the increased cell population with 4C DNA content, T521 treatment caused accumulation of mitotic markers in HeLa cells. Nocodazole blocks cell cycle at mitosis due to the disruption of microtubule-chromosome interaction, and western blot analysis showed an increase of protein levels of Plk1, Cyclin B1, and phospho-histone H3 (pHH-3) in nocodazole-treated HeLa cells. T521-treated HeLa cells also showed accumulation of these mitotic markers (Figure 3D), further supporting the conclusion that T521 induces mitotic delay in HeLa cells.

### T521 Induces defects in centrosome integrity, chromosome alignment and spindle assembly in HeLa cells

**Figure 4:**
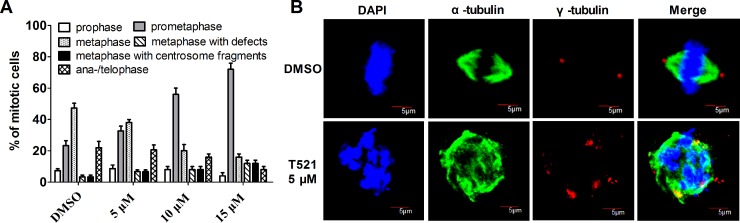
T521 induces defects in centrosome integrity, chromosome alignment and spindle assembly **A.** The distribution of mitotic phases within the total mitotic cells treated with or without T521. The percentage of mitotic cells with lagging chromosomes (metaphase with defects) or multiple centrosomes (>2) are also shown. Approximately 50 mitotic cells were analyzed each time and the results are the average from three independent experiments. Error bars represent SD. **B.** Representative Hela cells in metaphase treated with or without T521. HeLa cells were synchronized at the G_1_/S boundary by double-thymidine block and then released into fresh medium with DMSO or T521 (5 μM) for 10 hrs. Cells were fixed and stained for DNA (blue), α-tubulin (green) and centrosome (γ-tubulin, red). The images were taken using an Olympus FV1000 confocal microscopy. Here shows an untreated cell (DMSO) in metaphase as well as a T521-treated cell with condensed but randomly distributed chromosomes, a disorganized spindle, and multiple γ-tubulin foci. Scale bars represent 5 μm.

Plk1 is involved in multiple processes in mitosis. The inhibition of Plk1 activity leads to prometaphase arrest, and some condensed chromosomes are unable to align on the mitotic plate [37]. Overexpression of the PBD of Plk1 in HeLa cells triggers prometaphase arrest with mislocalized Plk1 and chromosome alignment defects, indicating the critical role of Plk1 localization in mitosis [38]. To test the effect of Plk1 PBD inhibitor T521 on mitosis, synchronized HeLa cells were released into fresh medium with T521 (5, 10, 15 μM) for 10 hrs. HeLa cells were stained with antibodies against α- and γ-tubulin as well as a DNA dye DAPI. We observed a prometaphase arrest in the presence of T521, and the arrest was more pronounced as T521 concentration increased (Figure 4A). The percentage of prometaphase cells increased from 23.3% in the control to 32.7%, 56.0%, and 76.2% in the presence of 5, 10, and 15 μM T521, respectively. A remarkable increase of metaphase cells with chromosome alignment defects was also observed. Moreover, cells with chromosome alignment defects also showed multiple γ-tubulin foci, indicating centrosome fragmentation (Figure 4A and 4B). These data support the conclusion that T521 inhibits Plk1 functions in cultured cells and causes significant mitotic defects. This effect of T521 on Plk1 likely contributes to the defects in centrosome maturation, spindle assembly and chromosome alignment after T521 treatment.

### Apoptosis in HeLa cells caused by T521 treatment

**Figure 5:**
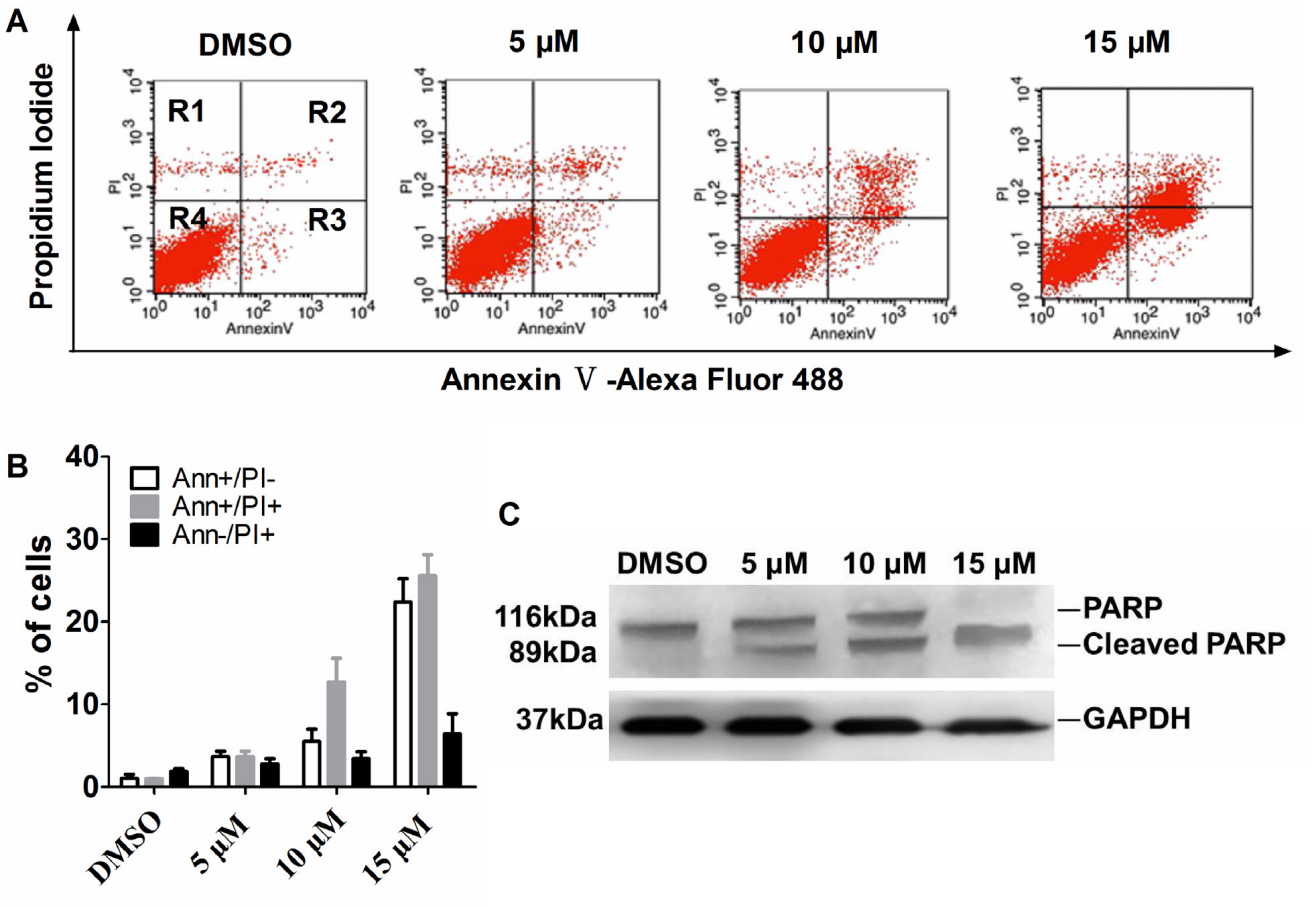
T521 induces apoptosis in HeLa cells **A.** Apoptosis caused by T521 treatment. Exponentially growing HeLa cells were treated with T521 at the indicated concentrations for 24 hrs and AnnexinV/PI dual-staining was performed. Apoptotic HeLa cells were analyzed by FACS. R1, AnnexinV-negative and PI-positive, dead cells; R2, double-positive, late apoptotic cells; R3, AnnexinV-positive and PI-negative, early apoptotic cells; R4, double-negative, live cells. **B.** Quantitative analysis of apoptotic cells in panel A (n=3). Error bars represent SD. **C.** PARP cleavage in T521-treated HeLa cells. HeLa cells were treated as described in A. Cell extracts were prepared and PARP cleavage was analyzed by western blotting. GAPDH served as the loading control.

Previous studies have shown that functional inhibition of Plk1 induces apoptosis in cancer cells [24], [37], [39], [40], [41]. To test whether apoptosis is also induced by T521, exponentially growing HeLa cells were treated with DMSO and various concentrations of T521 for 24 hrs, stained with Annexin V and Propidium Iodide (PI), and then analyzed by flow cytometry. Compared with DMSO control, T521 treatment led to an increase of cell population positive for Annexin V staining, indicative of apoptosis (Figure 5A and 5B). The appearance of cleaved PARP (Poly[ADP-ribose]-polymerase-1) is a notable marker for Caspase-3 activation and late apoptosis [42]. Western blot analysis demonstrated an increase of PARP cleavage induced by T521, further confirming apoptosis (Figure 5C). Taken together, these results suggest that T521 induces apoptosis in HeLa cells, presumably by functional disruption of Plk1.

### T521 inhibits tumor growth in nude mice

**Figure 6:**
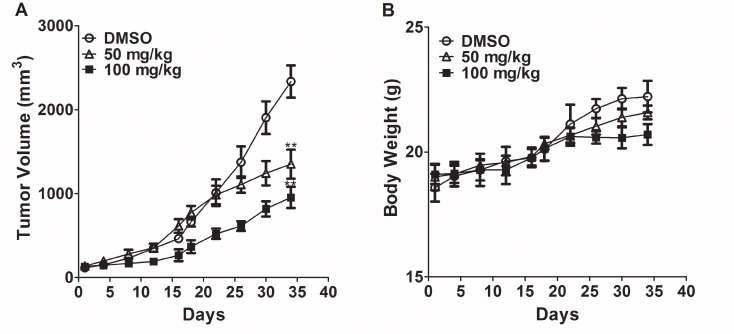
T521 suppresses tumor growth **A.** Nude mice bearing established xenografts of A549 (n=10 for each group) were intratumorally treated with DMSO, T521 (100 and 50 mg/kg) on Wednesdays and Fridays for 5 weeks. The tumor size over time is displayed. **p<0.01 compared to DMSO. p values were calculated using t test. Error bars represent SD. **B.** The total body weight of individual mice in the three groups was determined twice per week and the average body weights are plotted.

We next assessed the *in vivo* anticancer activity of T521 in nude mice xenografted with A549 lung-carcinoma cells [37]. The nude mice were treated with T521 by intratumoral injection at 50 and 100 mg/kg on Wednesdays and Fridays for 5 weeks. Tumor volumes were determined with a caliper on Monday and Thursday during drug administration cycle. An obvious reduction of tumor volume was observed after 5 weeks of treatment with T521 compared with vehicle DMSO treatment (Figure 6A). In contrast, the difference of body weight between T521-treated and control nude mice was much less significant, indicating that T521 is well tolerated (Figure 6B). These results demonstrate the anticancer activity of T521, presumably by inhibiting Plk1 functions.

## DISCUSSION

Cancer cells display characteristics of sustaining proliferative signaling, evading growth suppressors, and resisting cell death [43], and Plk1 is involved in all these processes. Thus, Plk1 is an appealing therapeutic target for cancer treatment, and a number of Plk1 inhibitors are currently under preclinical or clinical trials [7]. However, the lack of specificity for ATP-competitive Plk1 inhibitors is a major concern because of the widely conserved kinase domain of Plks and other kinases. In addition, drug resistance is commonly associated with these Plk1 inhibitors [22]. The PBD is unique to Plks family, and required for Plk1's subcellular localization for its mitotic function, thus the PBD represents an attractive target for Plk1 specific inhibitors. Some peptide inhibitors of Plk1 PBD have been explored [44–47], but their poor stability and low permeability prevent further clinical application [48]. Therefore, we were encouraged to discover additional Plk1 PBD inhibitors, which could be used for anti-Plk1 cancer therapy.

In this study, we adopted a FP-based high-throughput screening system using FITC-Poloboxtide, an optimal PBD-binding phosphopeptide [5], for the identification of small-molecule inhibitors of Plk1 PBD. From about 20,000 molecules, T521 was identified. T521 likely inhibits Plk1 activity by binding to its PBD based on the following observations. First, T521 significantly blocks the interactions between Plk1 PBD and FITC-Poloboxtide or its substratemimic GST-Map205^PBM^ *in vitro*. Moreover, T521 reduces Plk1-Bub1 interaction *in vivo*. Furthermore, using HPLC-Q-TOF MS analysis, our data indicate that the binding between T521 and Plk1 PBD is likely covalent. Importantly, unlike other known PBD inhibitors, T521 inhibits the PBD of Plk1, but not those of Plk2-3, indicating its specificity toward Plk1. Therefore, we identified a novel Plk1 PBD inhibitor with the desirable specificity to Plk1. To our knowledge, this is the first report for a compound with phenylsulfonyl oxazole scaffold that shows anti-Plk1 activity. It is our future interest to synthesize more potent Plk1 PBD inhibitors using this compound as a lead.

Our observations support the notion that the interaction between T521 and Plk1 PBD is covalent. After incubation of Plk1 with T521, the mass of Plk1 protein increase indicates that three T521 fragments are added. Protease digestion of the Plk1-T521 mixture followed by Nano-LC-LTQ-Orbitrap XL MS analysis identified three covalent labeling sites in Plk1, one in the kinase domain (K209), and two in the PBD (K388 and K574). It is possible that T521 undergoes a nucleophilic reaction with Plk1. First, the carbon of oxazole linking ethylsulfonyl group in T521 shows more cationic (δ+) and is easy to be attacked by nucleophiles, Moreover, the ethylsulfonyl group of T521 is a perfect leaving group. Therefore, after attacking T521 by the electron-rich alpha amino (alpha NH_2_) of K or R in Plk1, covalent addition of T521 to Plk1 could happen (Supplementary Figure S4). Surprisingly, the covalent modification of Plk2-3 by T521 was not detected, which could explain the specificity of T521 toward Plk1, but it is unclear why T521 interacts specifically with Plk1. One possibility is that the unique structure or the amino acid residues in Plk1 PBD allow the access of T521 to the lysine residues for a nucleophilic reaction, which might in part contribute to the specificity.

Because the lysine residues that show covalent binding with T521 are not located within the phosphopeptide binding pocket of Plk1 PBD, one critical question is how the interaction between T521 and Plk1 PBD interferes with the functions of Plk1. It is possible that the covalent binding of T521 to the Plk1 lysine residues does not directly block the peptide association of Plk1 PBD. Instead, the changes in the secondary structure of Plk1 PBD induced by T521 binding block its peptide association, thereby interfering with Plk1 functions. Indeed, the CD spectra of T521/Plk1 PBD complex and native Plk1 PBD differ drastically, indicating the secondary structure changes of Plk1 PBD after its covalent binding to T521. However, a crystallographic structure analysis of T521/Plk1 PBD complex is required to validate the specific binding between T521 and Plk1 PBD and its effect on Plk1 PBD structure.

Plk1's subcellular localization is important for its mitotic functions. The Plk1-Bub1 interaction recruits Plk1 to kinetochores, which facilitates the kinetochore localization of BubR1, Mad2, and other checkpoint components [35]. Moreover, the kinetochore localization of Plk1 allows the phosphorylation of BubR1 by Plk1, which in turn enhances the stability of kinetochore-microtubule interactions [49]. Our data show that T521 reduces Plk1-Bub1 binding *in vivo*, which will decrease the kinetochore recruitment of Plk1 and destabilize of kinetochore-microtubule interactions. In support of this speculation, HeLa cells treated with T521 showed multiple mitotic defects, including centrosome fragmentation, abnormal chromosome alignment, and spindle assembly defect. All the evidence suggests that Plk1 PBD is likely the *in vivo* target of T521.

Taken together, we have identified a new Plk1 inhibitor that disrupts the PBD functions of Plk1. Moreover, T521 delays cell cycle progression at mitosis and causes multiple mitotic defects presumably by disrupting PBD functions of Plk1. Because T521 has a scaffold different from all other Plk1 PBD inhibitors, it could be an attractive lead compound for a new group of Plk1 PBD inhibitors.

## MATERIALS AND METHODS

### Synthesis of T521

We identified T521 as a potent Plk1 PBD inhibitor from the diverse chemical libraries of 20,000 small-molecule compounds (ChemDiv). Because a large amount of T521 was needed for its characterization, we designed chemical synthesis of T521. The protocol for the synthesis of T521 was described in the SUPPLEMENTARY MATERIALS AND METHODS, and the purity is more than 97.0%.

### Cell culture

Human tumor and normal cell lines were obtained from American Type Culture Collection and were not authenticated by the authors for this study. The tissue culture medium for each cell line was used as recommended by suppliers.

### Fluorescence polarization-based high-throughput screening system

FP assay was performed as follow. Briefly, 30 μL Plk1 PBD proteins (400 nM) were incubated with 0.3 μL tested compounds (1 mg/mL) with shaking for 1 hr at room temperature (RT) prior to addition of 30 μL FITC-Poloboxtide (FITC-GPMQSpTPLNG-OH; λex/λem: 485/535 nm; 60 nM) in FP buffer (50 mM Tris-HCl, 10 mM NaCl, 1 mM EDTA pH8.0). Three wells containing 30 μL FITC-Poloboxtide (60 nM) and 30 μL Plk1 PBD (400 nM) were used as negative control (0% inhibition); three wells containing 60 μL FITC-Poloboxtide (30 nM) only were used as positive control (100% inhibition). Fluorescence polarization was analyzed after incubation of FITC-Poloboxtide for 15 min in a 384-well black opaque microplate (Cat# 6007270, PerkinElmer) using Envision Multilabel Reader (PerkinElmer) at 25°C. We calculated the percentage of inhibition for each sample as follows:

$$\text{Inhibition}(\%)=\frac{\text{Negative}-\text{Sample}}{\text{Negative}-\text{Positive}}\times 100\%$$

We plotted the inhibition values and determined IC_50_ using GraphPad Prism5.0. Together with the FITC-labeled peptides used for Plk1 PBD as mentioned above, the following peptides were used for specificity assays, FITC-GPMQTSpTPKNG-OH (Plk2 PBD), FITC-GPLATSpTPKNG-OH (Plk3 PBD). As described above for the FP assay, proteins were used at the following final concentrations: Plk1 PBD, 200 nM; Plk2 PBD, 450 nM; Plk3 PBD, 600 nM. All FITC-labeled peptides were chemically synthesized (China Peptides Co., Ltd., Shanghai) and further verified by mass spectrometry, and the purity is more than 95.0%.

### ELISA-based PBD binding inhibition assay

GST-Map205^PBM^ proteins (10 μg/mL and 100 μL/well) were immobilized onto a 96-well plate (Cat#3590; Corning) in 1×coating buffer (0.1 M NaH_2_PO_4_, 0.15 M NaCl pH6.5) at 37°C for 2 hrs. The wells were washed 3 times with PBST (PBS with 0.1% Tween 20) and incubated with 200 μL of blocking buffer (PBST with 10% Milk). Plk1 PBD proteins with His-tag (3 μg/mL) were incubated with T521 at several concentrations (two-fold dilutions and the initial concentration is 400 nM, 8 samples total) for 45 min at RT prior to the addition of the mixture into a Map205^PBM^-coated plate. After incubation, the plate was rinsed 3 times with PBST, and then probed for 45 min at 37°C with mouse anti-His primary antibody (Cat# sc-8036; Santa Cruz; 1:2000; 100 μL/well), followed by 3 washes with PBST. The plate was further probed for 45 min at 37°C with goat anti-mouse IgG-HRP secondary antibody (Cat# sc-2005; Santa Cruz; 1:2000; 100 μL/well). After 3 washes with PBST, the plate was incubated for 5 min at RT with 3,3′, 5,5′-tetramethylbenzidine (TMB) solution (Cat# PA-107; TianGen Biotech; 100 μL/well) in the dark. The reaction was stopped by the addition of 2 M H_2_SO_4_ (50 μL/well) and the OD_450_ was immediately measured by Envision Multilabel Reader (PerkinElmer).

### Co-immunoprecipitation (IP) assay

HeLa cells were synchronized in G_1_/S boundary by double-thymidine block as described [50], and then released into medium containing DMSO and T521 (5, 10, 15 μM) for 10 hrs. For Co-IP assay, rabbit polyclonal anti-Bub1 antibody (Cat# ab70372; Abcam) was coupled to Protein A-Agarose beads (Cat# sc-2001; Santa Cruz) at a concentration 100 μg/mL. Treated HeLa cells were lysed with RIPA buffer. After clearing by centrifugation, the lysate was incubated with the beads coupled to anti-Bub1 antibody for 2 hrs at 4°C. The beads were washed with the lysis buffer for three times. The proteins bound to the beads were dissolved in loading buffer and separated by 10% SDS-PAGE, and probed with the mouse monoclonal anti-hPlk1 antibody (Cat# P5998; Sigma; 1:3000) and rabbit polyclonal anti-Bub1 antibody (Cat# ab70372;Abcam; 1:2000), respectively.

### The assay of the inhibition of Plk1 PBD by T521

For the temperature inhibition assay, 30 μL Plk1 PBD (400 nM) was incubated with T521 (two-fold dilutions and the initial concentration is 400 nM, 8 reactions total, 0.3 μL/well) for 1 hr at indicated temperatures, and 30 μL FITC-Poloboxtide (60 nM) was added into the reaction mixtures. After 15 min, mP value was measured, which is used to plot the inhibition curve and determine the IC_50_ using GraphPad Prism5.0.

To assess the inhibition of Plk1 PBD by T521 over time, 30 μL Plk1 PBD (400 nM) was incubated with T521 at different concentrations (two-fold dilutions and the initial concentration is 400 nM, 8 reactions total, 0.3 μL/well) for the indicated times at RT. Then 30 μL FITC-Poloboxtide (60 nM) was added into the reaction mixtures. After 15 min, the mP value was measured, which is used to plot the inhibition curves and determine the IC_50_. All experiments were performed in triplicate.

### T521/Plk1 PBD binding kinetics assay

For T521/Plk1 PBD binding kinetics assay, 200 μM, 400 μM and 800 μM of T521 (0.3 μL/well) was incubated with 30 μL Plk1 PBD (400 nM) for the indicated times at RT and 30 μL FITC-Poloboxtide (60 nM) was added. After 15 min, mP value was measured to determine the inhibition effect as described above. Three independent experiments were performed.

### The MTT assay

The MTT assay was performed as described [51]. In brief, cells were seeded at 3000~5000 cells/well into 96-well plates (200 μL/well) and incubated overnight. We replaced the growth medium with fresh medium containing T521 at several concentrations (two-fold dilutions and the initial concentration is 50 μM, 10 reactions total). After further incubation for 48 hrs in the presence of T521, the medium was removed from all wells and the cells were incubated with 200 μL of fresh medium for additional 24 hrs. We then added 22 μL of MTT solution (Amresco; 5 mg/mL in PBS) to each well and incubated the plate for 4 hrs at 37°C. After removing the medium, we dissolved the purple MTT-formazan crystals by adding 150 μL of DMSO to each well and recorded absorbance at 560 nm by Envision Multilabel Reader (PerkinElmer). The inhibition curve was plotted to determine the IC_50_ of various cell lines using GraphPad Prism5.0.

### Cell cycle analysis

HeLa cells (1.0×10^5^ cells/well) were synchronized at G_1_/S boundary by double-thymidine block and then released into various media with indicated reagents. For cell cycle analysis, HeLa cells were harvested at different time points, washed twice with PBS, fixed in ice-cold 70% ethanol for 30 min at 4°C, and incubated with 500 μL of Propidium Iodine (Sigma; 10 μg/mL) and RNase A (Sigma; 1 mg/mL) for 30 min at 37°C in the dark prior to flow cytometry analysis (FACScan, BD Biosciences).

### Apoptosis assay

For apoptosis assay, HeLa cells were seeded at 3.0×10^4^ cells/well into 6-well plates and incubated overnight. Then exponentially growing HeLa cells were treated with T521 at the indicated concentrations for 24 hrs and Annexin V/PI dual-staining was carried out using a commercial apoptosis assay kit (Beijing 4A Biotech. Co., Ltd.). DMSO-treated cells were taken as control.

### Western blot assay

HeLa cells were collected after trypsinization and lysed with RIPA buffer. Equal amount of proteins (30 μg/lane) were loaded, separated by 15% SDS-PAGE. After blocking with blocking buffer for 2 hrs at RT, the membrane was incubated with the following primary antibodies diluted in blocking buffer overnight at 4°C: anti-hPlk1 mouse monoclonal antibody (Cat# P5998; Sigma; 1:3000), anti-CyclinB1 rabbit polyclonal antibody (Cat# SAB4503501; Sigma; 1:2000), anti-pHH3 rabbit monoclonal antibody (Cat# 04-817; Millipore; 1:2000), anti-GAPDH rabbit polyclonal antibody (Cat# P30008; Abmart; 1:2000). The membrane was washed 3 times and then incubated with Horseradish Peroxidase-conjugated secondary antibodies for 1 hr at RT and visualized with ECL system (Millipore). The level of GAPDH was used as the loading control.

We also performed a western blot assay to examine PARP cleavage, and the protocol was similar as describe above. Equal amount of proteins (30 μg/lane) were loaded, separated by 12% SDS-PAGE. Anti-PARP rabbit polyclonal antibody (Cat# 9542P; Cell Signaling Technology; 1:1500) was used as primary antibody. GAPDH was used as the loading control.

### Immunofluorescence assay

To determine mitotic phase distribution, about 50 mitotic cells were analyzed. Cells were classified according to the structure and alignment of the chromosomes as well as the number and location of the centrosomes. All experiments were performed in triplicate. For confocal microscopy, HeLa cells grown on coverslips were fixed and permeabilized in methanol for 15 min at −20°C and then incubated with blocking buffer (PBS with 0.1% Triton X-100 and 5% BSA) for 2 hrs at RT. And then the coverslips were followed by washing and incubation with primary antibodies and fluorophore-conjugated secondary antibodies for 1 hr at RT, respectively. All antibodies were diluted in blocking buffer and the coverslips were washed three times with PBST, and then all coverslips were treated with 4',6-diamidino-2-phenylindole (DAPI) (Sigma; 2 μg/mL) for 2 min at RT, washed again and mounted onto slides using ProLong Diamond Antifade Mountant (Cat# 36961; Molecular Probes). Immunofluorescence images were taken with Olympus FV1000 Confocal Laser Microscope (Olympus, Japan) at 600×magnification. All images were processed using FV10-ASW 3.0 Viewer Software System (Olympus, Japan). The following antibodies were used: anti-α-Tubulin mouse monoclonal antibody (Cat# T9026; Sigma; 1:800), anti-γ-Tubulin rabbit polyclonal antibody (Cat# T3559; Sigma; 1:1500), Alex Fluor 488-conjugated goat anti-mouse IgG (Cat# A-11011; Molecular Probes; 1:1000) and Alex Fluor 594-conjugated donkey anti-rabbit IgG (Cat# A-21207; Molecular Probes; 1:1000).

### Analysis of the anticancer activity of T521 in mice

Female athymic nude mice were injected with 2.0 × 10^7^ A549 lung-carcinoma cells subcutaneously. After 14~20 days, when the tumor volumes reached 100~150 mm^3^, animals were divided into treatment (T521; 50, 100 mg/kg) and control (DMSO) groups with ten mice in each group. T521 (0.2 g/mL) dissolved in DMSO was administered alone and mice were treated with T521 by intratumoral injection at the different injection points according to indicated dose on Wednesdays and Fridays for 5 weeks. Tumor volumes were determined with a caliper on Monday and Thursday during drug administration cycle. Tumor volume (mm^3^) was calculated using the following equation: V=L×S^2^×π/6, where L is the length and S is width of the tumor. The body weight of these mice was determined as an indicator of tolerability on the same days. Student's t-tests were performed for statistical evaluation among treatment and control groups. All mice were properly treated in accordance with the guidelines of the local animal committee.

## MATERIALS AND METHODS


