# Supplementary material for: Identification of a novel Polo-like kinase 1 inhibitor that specifically blocks the functions of Polo-Box domain
Source: Oncotarget. 2016 Nov 25;8(1):1234–46. doi: 10.18632/oncotarget.13603 (PMC5352051; doi:10.18632/oncotarget.13603)
Supplement: Supplementary file 1 [file oncotarget-08-1234-s001.pdf]

Identification of a novel Polo-like kinase 1 inhibitor that specifically blocks the functions of Polo-Box domain

MATERIALS AND METHODS

Chemical syntheses of T521[a]

Experiments

Chemistry

^{1}H NMR spectra were recorded on Bruker spectrometer at 400 MHz. The purity of **T521** was determined by LC-MS using Agilent 1100 HPLC system. Commercially available reagents and solvents were purchased from Sigma-Aldrich and were used for chemical synthesis of T521.

4-fluoro-N-(2,2,2-trichloro-1-hydroxyethyl) benzamide (3)

A mixture of 4-fluorobenzamide **1** (13.90 g, 100 mmol) and chloral hydrate **2** (19.70 g, 120 mmol) was heated at 95°C for 1.5 hrs. The reaction mixture was cooled down to the RT (room temperature), washed with cold chloroform and filtered. The solid was re-crystallized from ethanol-water to afford **3** (26.16 g, yield 92%) as a white solid.

^{1}H NMR (400 MHz, CD_3OD) δ 7.92−7.94 (m, 2H), 7.22−7.24 (m, 2H), 6.12 (s, 1H). MS(ESI): *m/z*, 285.9 $[M+H]^+$.

4-fluoro-N-(1,2,2,2-tetrachloroethyl)benzamide (4)

To a stirred suspension of chloralamide **3** (7.30 g, 26 mmol) in 120 mL of dry chloroform a suspension of phosphorus pentachloride (5.41 g, 26 mmol) in 40 mL of dry chloroform was slowly added at 20~25°C. The mixture was allowed to stir at room temperature for 4 hrs. The solution was concentrated under reduce pressure to afford a yellow solid. The crude product was washed with cold petroleum ether to give **4** (6.90 g, purity 60%, yield 53%) as a white solid without any other purification.

MS(ESI): *m/z*, 299.9 $[M-3]^+$.

N-(2,2-dichloro-1-(phenylsulfonyl)vinyl)-4-fluorobenzamide (5)

To a solution of chloralkane **4** (0.75 mg, 2.48 mmol) in 15 mL of dry acetonitrile sodium benzenesulfinate (0.82 g, 4.96 mmol) was added. The reaction mixture was allowed to stir at 70°C. After 3 hrs, the precipitate was filtered off and the filtrate was evaporated under reduce pressure. The residue was dissolved in 15 mL of tetrahydrofuran and triethylamine (0.7 mL, 4.96 mmol) was added to the solution at 0°C. The mixture was warmed

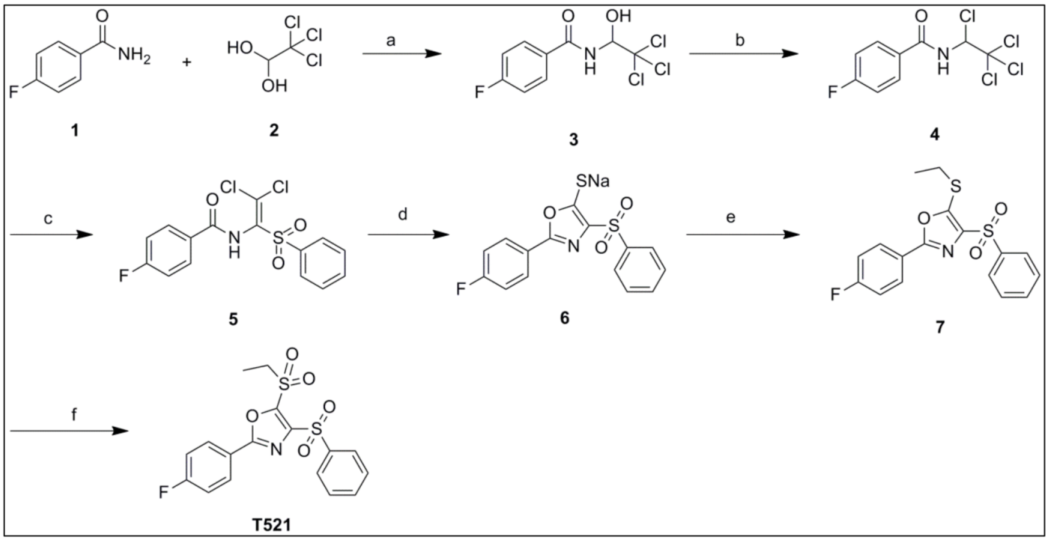

[a]**Reagents: a.** 95°C, 1.5 hrs. **b.** PCl_5, $CHCl_3$, 25°C, 4 hrs. **c.** (1) Sodium benzenesulphinate, CH_3CN, 70°C, 3 hrs; (2) TEA, THF, 0~10°C, 12 hrs. **d.** NaHS, 25°C, 18 hrs. **e.** Iodoethane, 25°C, 24 hrs. **f.** H_2O_2, acetic acid, reflux, 1 hr.

to room temperature and stirred for further 12 hrs. The precipitate was filtered off and the filtrate was evaporated under reduce pressure. The crude product was purified by silica column chromatography eluting with petroleum ether/ethyl acetate (5/1, V/V) to afford **5** (0.69 g, yield 74%) as a white solid.

^{1}H NMR (400 MHz, DMSO-d_6) δ 10.71 (s, 1H), 8.03−8.07 (m, 2H), 8.01−8.03 (m, 2H), 7.78−7.81 (m, 1H), 7.68−7.71 (m, 2H), 7.39−7.42 (m, 2H). MS(ESI): *m/z*, 374.0 $[M+H]^+$, 395.9 $[M+Na]^+$.

5-(ethylthio)-2-(4-fluorophenyl)-4-(phenylsulfonyl)oxazole (7)

To a solution of sodium hydrosulfide (140 mg, 2.49 mmol) in 10 mL of ethanol amide **5** (310 mg, 0.83 mmol) was added at 10~15°C. The mixture was allowed to stir at 25°C for 18 hrs. The precipitate was filtered off, and the filtrate was added with iodoethane (130 mg, 0.83 mmol). The mixture was stirred at 25°C. After 24 hrs, the precipitate was filtered off and the filtrate was evaporated under reduce pressure. The residue was purified by silica column chromatography eluting with petroleum ether/ethyl acetate (8/1, V/V) to afford **7** (552 mg, yield 56%) as a white solid.

^{1}H NMR (400 MHz, DMSO-d_6) δ 7.97−8.01 (m, 4H), 7.75−7.77 (m, 1H), 7.66−7.70 (m, 2H), 7.37 (m, 2H), 3.23 (*q*, 2H, J = 7.2 Hz), 1.33 (*t*, 3H, J = 7.2 Hz). MS(ESI): *m/z*, 364.1 $[M+H]^+$.

5-(ethylsulfonyl)-2-(4-fluorophenyl)-4-(phenylsulfonyl)oxazole (T521)

A mixture of oxazole **7** (726 mg, 2 mmol), 10 mL of acetic acid and 3mL of hydrogen peroxide (30% in water) was refluxed for 1 hr. The reaction was cooled down to RT. The solvent was removed *in vaccum*. The residue was treated with ethanol and the precipitate was filtered off, washed with ethanol. The crude product was crystallized from ethyl acetate to afford **T521** (387 mg, purity 97%, yield 48%) as a white solid.

^{1}H NMR (400 MHz, DMSO-d_6) δ 8.09−8.11 (m, 2H), 8.04−8.06 (m, 2H), 7.81−7.84 (m, 2H), 7.72−7.75 (m, 2H), 7.43−7.46 (m, 2H), 3.80 (*q*, 2H, J = 6.0 Hz), 1.31 (*t*, 3H, J = 6.0 Hz).^{13}C NMR (100 MHz, DMSO-d_6) δ 165.70, 163.9, 161.34, 146.76, 143.79, 138.17, 135.19, 130.31, 130.23, 129.85, 128.46, 121.16, 121.13, 116.86, 116.68, 50.40, 6.25. HRMS (ESI), *m/z*: calcd for $C_{17}H_{14}FNO_5S_2$ $[M + H]^+$: 396.0370, found: 396.03887. HPLC purity: 97.00%.

Plasmid construction and protein expression

The C-terminal DNA fragments of human Plk1 (326-603 aa), Plk2 (355-685 aa), and Plk3 (335-646 aa) that cover the Polo-Box domain were amplified by PCR from Plk1 plasmid, HeLa cell cDNA and HepG2 cell cDNA, respectively. The PCR products of Plk1 PBD were inserted into pET-28a vector carrying a 6×His tag at both N and C-termini, respectively. The PCR products of Plk2 PBD and Plk3 PBD were ligated into pGEX-4T-1 vector carrying an N-terminal GST tag. Proteins were expressed in *E.coli* Rosetta BL21(DE3) after induction with 0.5 mM IPTG at 20°C overnight and purified by HisTrap™ HP (GE Healthcare) or GST-affinity chromatography. Plk1-3 PBD proteins were dialyzed in a buffer containing 20 mM Tris-HCl (pH8.0), 100 mM NaCl, 1 mM EDTA, 10% glycerol.

The DNA fragment encoding *Drosophila* Map205^{PBM} (276-325 aa) was synthesized by Beijing Ruibo Biotechnology Company and then directly cloned into pGEX-4T-1 vector carrying an N-terminal GST tag. Map205^{PBM} proteins were expressed in *E.coli* BL21(DE3) cells with 0.5 mM IPTG at 37°C for 4 hrs, and were purified by GST-affinity chromatography. GST-Map205^{PBM} proteins were dialyzed in a buffer containing 20 mM Tris-HCl (pH8.0), 100 mM NaCl, 1 mM EDTA. The concentrations of all recombinant proteins were determined using BCA™ Protein Assay Kit (Thermo Fisher) according to the manufacturer's instruction.

HPLC-Q-TOF-mass spectrometry analysis

To analyze the covalent interaction between T521 and Plk1 protein, 10 µM Plk1 was incubated with or without T521 (500 µM) in 50 mM Tris-HCl (pH8.0) at RT for 30 min. For each assay, 5 µg protein was injected into a prepacked reverse-phase column for HPLC (Zorbax 300SB-C8; 4.6×250 mm; 5 µm; Agilent). After desalting, the sample (4 µL) was eluted with a gradient program (A=0.1% FA (formic acid) in water, B = 0.1% FA in acetonitrile (ACN); flow rate=1 mL/min; HPLC gradient program: 0-5 min, 5% B; 5-10 min, 5-50% B; 10-15 min, 50-90% B; 15-16 min, 90-5% B; 16-21 min, 5% B). The samples were eluted into a Q-TOF mass spectrometer (Agilent, USA). Data were acquired in profile mode scanning m/z 600-1800. Mass spectra were deconvoluted using Mass Hunter software (Agilent), and maximum entropy was performed for deconvolute algorithm. All experiments were performed in triplicate. As described above for the MS assay, the reaction ratio (Plk1-3/T521) was kept at 1:50. Plk1, Plk2 and Plk3 proteins were purchased from Sino Biological Inc.

Protease digestion of peptide fragments and Nano-LC-LTQ-Orbitrap XL MS analysis

In-gel proteolytic digestion of peptides was performed as described. Briefly, the sample (T521/Plk1 covalent product) was loaded, separated by 10% SDS-

PAGE, and then the stained band was excised from the polyacrylamide gel, washed twice with water, and destained with 40% ACN/50 mM NH_4HCO_3. The gel pieces were dehydrated with 100% ACN and dried for 5 min using a speedvac. Disulfide bonds were reduced by treatment with DTT (10 mM, 56°C, 45 min), and the free sulfhydryl groups were alkylated with iodoacetamide (55 mM, 25°C, 60 min in the dark). After drying with a speedvac, the gel was rehydrated using 100 ng/µL trypsin (50 mM NH_4HCO_3, pH8.3) on ice for 30 min, and the digestion was carried out at 37°C for 60 min and then quenched with 1.0% FA. The tryptic peptides were extracted twice with 60% ACN containing 0.1% FA, and then the combined digest solution was concentrated to 25 µL under vacuum.

To identify the sites of covalent binding in Plk1, the tryptic fragments were loaded onto a nano-HPLC system equipped with a self-packed reverse-phase column (150 µm×3 cm, 5 µm C18) and a nano column (75 µm×15 cm, 3.0 µm C18) coupled online to a LTQ-Orbitrap XL mass spectrometer (Thermo Fisher Scientific, USA). The protein sample was eluted with an HPLC gradient program (0%–100% B in 100 min, A = 0.5% FA in water, B = 0.5% FA in ACN, flow rate = 300 nL/min). The gradient elution program was as follows: 0–3 min, 4%–10% B; 3–78 min, 10%–36% B; 78–83 min, 36%–80% B; 83–90 min, 80% B; 90–91 min, 80%–4% B; 91–100 min, 4% B. Eluting peptide cations were converted to gas-phase ions by Nanospray Flex ion source with 2.1 kV. Survey full scan MS spectra were acquired from m/z 300 to m/z 1,800, and the 10 most intense ions with charges more than 2 and above an intensity threshold 500 were fragmented in the linear ion trap using normalized collision energy of 35%.

The raw data was processed using Proteome Discoverer software (Thermo Fischer). MS2 spectra were searched with SEQUEST engine against target Plk1 protein and common pollution proteins database (246 Protein Sequences). Database searches were performed with the following parameters: precursor mass tolerance 20 ppm; MS/MS mass tolerance 0.6 Da; two missed cleavage for tryptic peptides; variable modifications oxidation (M), T521 fragment modification (+301.02) (K, R), fixed modification Carbamidomethyl (C), the results were filtered for a 1% false discovery rate (FDR) at the PSM level utilizing the percolator-based algorithm. Peptide identifications were grouped into proteins according to the law of parsimony.

Circular dichroism spectroscopy

T521 was dissolved in acetonitrile. All samples including Plk1 PBD and Plk1 PBD/T521 mixture were prepared as previously described. Circular dichroism (CD) spectra in the far UV region (200–260 nm) were carried out at ambient temperature on a Chirascan-plus Circular Dichroism Spectrometer (Applied Photophysics Ltd, UK). Three scans were collected and averaged at a scan speed of 120 nm/min, with a time constant of 1 s and band width of 1 nm. A mean residue molecular weight of 110 was used in the calculation of mean residue ellipticity. Raw data were processed by smoothing and subtraction of buffer spectra, according to the manufacturer's instructions. Three independent experiments were performed for each sample. Spectral deconvolution of CD data was performed using the CDNN to determine relative quantities of α-helix, β-sheet, β-turn and random coil.

SUPPLEMENTARY FIGURES

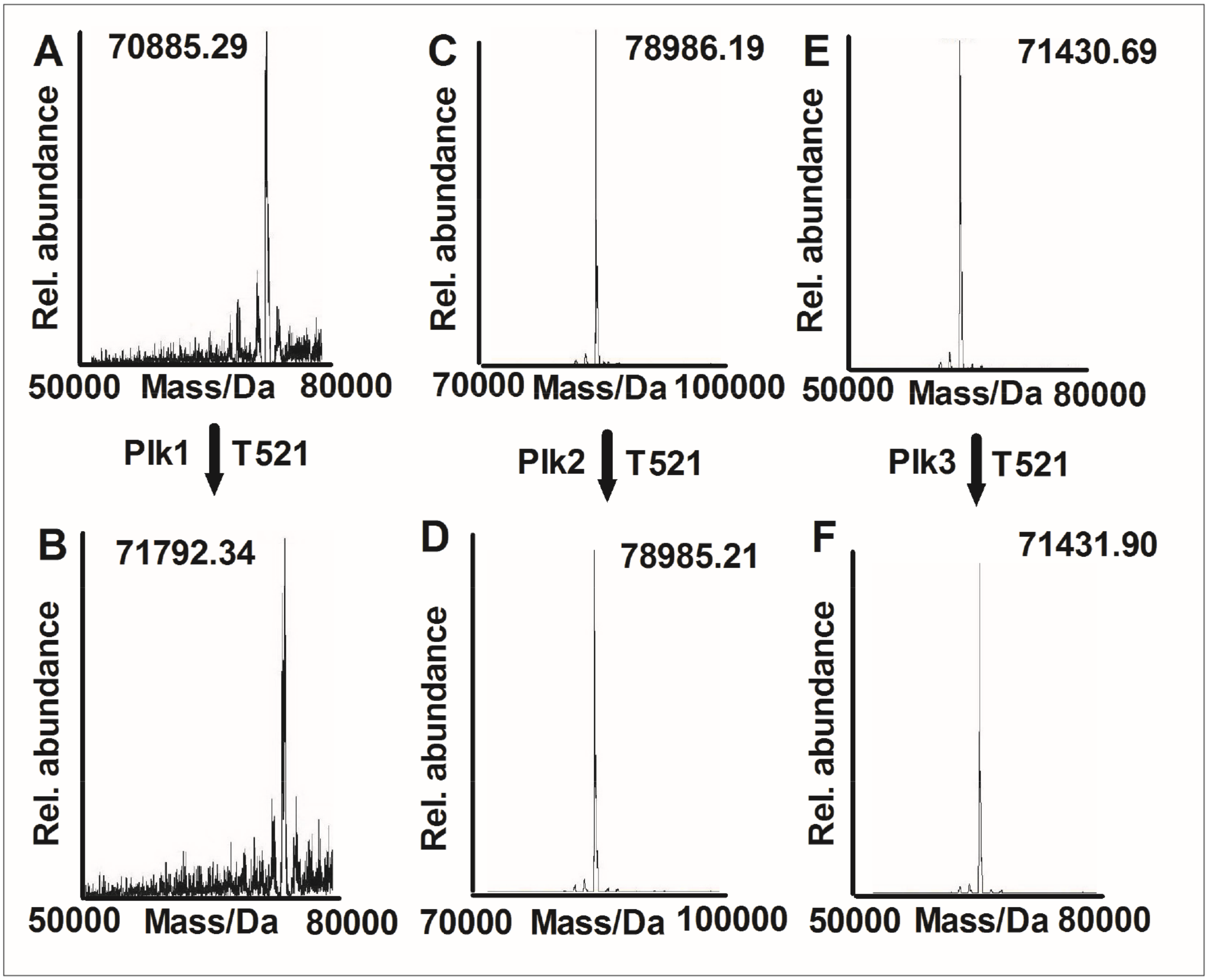

Supplementary Figure S1: Analysis of binding mode between T521 and Plk1-3 using HPLC-Q-TOF MS. Plk1-3 (10 µM) proteins were incubated with or without T521 (500 µM) in 50 mM Tris-HCl (pH8.0) at RT for 30 min. The desalted samples (5 µg) were eluted with an HPLC gradient program into a Q-TOF mass spectrometer (Agilent, USA). Mass spectra were obtained after deconvolution. **A, C, E.** The analysis of Plk1-3 proteins after incubation in the absence of T521. **B, D, F.** The analysis of Plk1-3 proteins after incubation with T521.

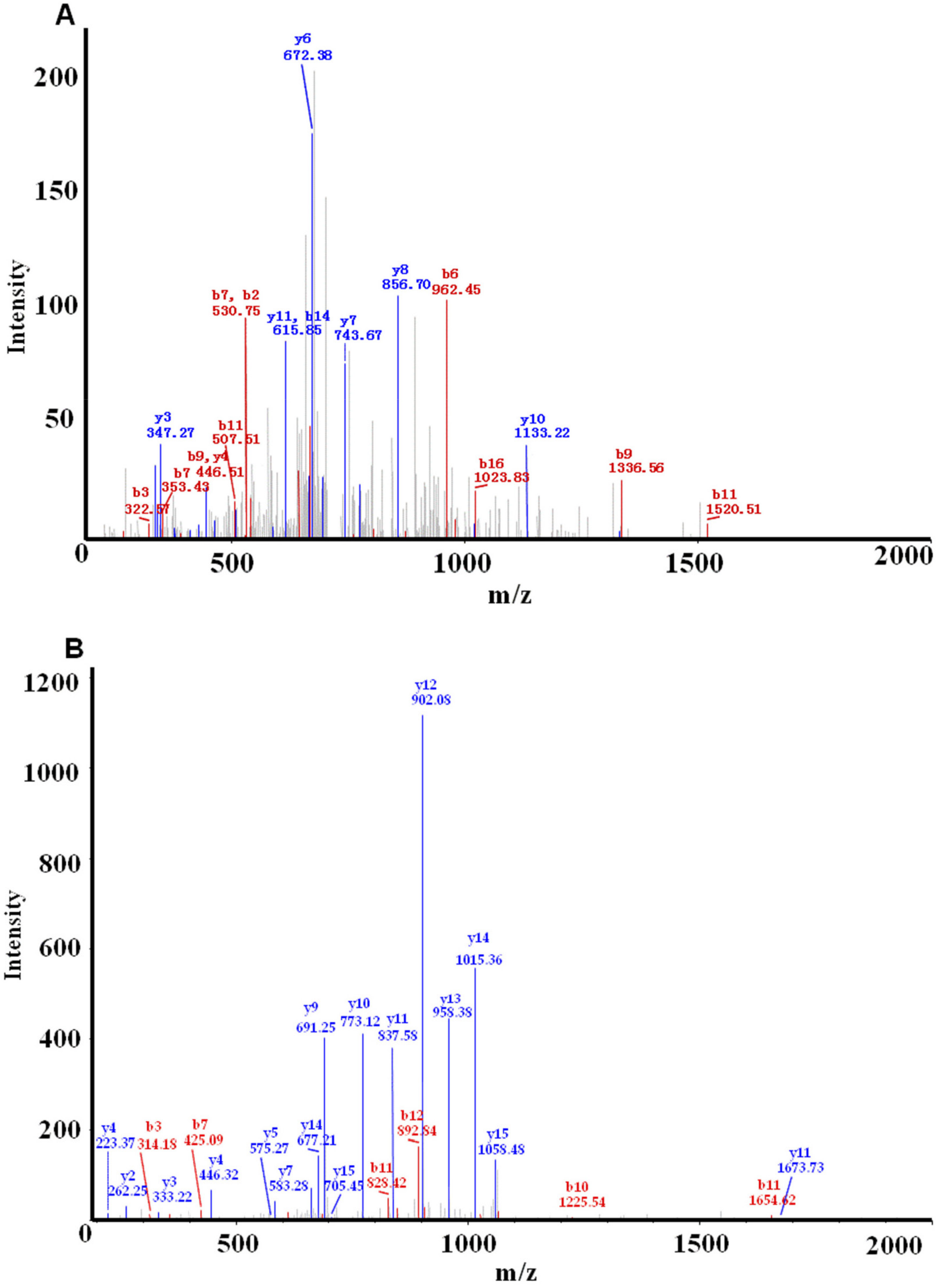

Supplementary Figure S2: Nano-LC-LTQ-Orbitrap XL MS analysis after protease digestion of peptide fragments. Ions of type b and y are shown in red and blue, respectively. A. Peptide KTLCGTPNYIAPEVLSK (Plk1 residues 209-225 aa). **B.** Peptide LSLLEEYGCCKELASR (Plk1 residues 564-579 aa).

(*Continued*)

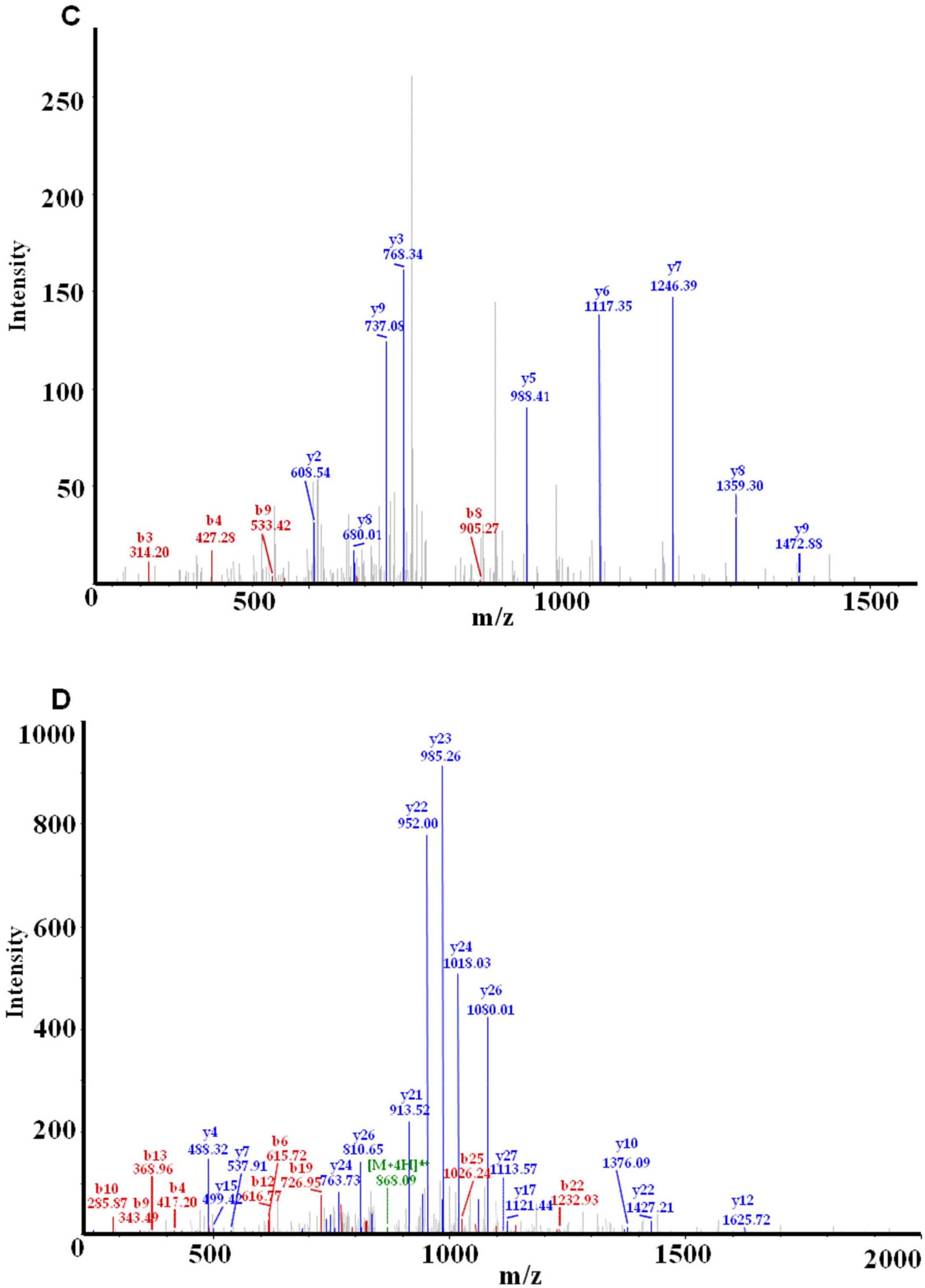

Supplementary Figure S2: (*Continued*) C. Peptide LSLLEEYGCCK (Plk1 residues 564-574 aa). **D.** Peptide CHLSDMLQQLHS VNASKPSER (Plk1 residues 372-392 aa).

(*Continued*)

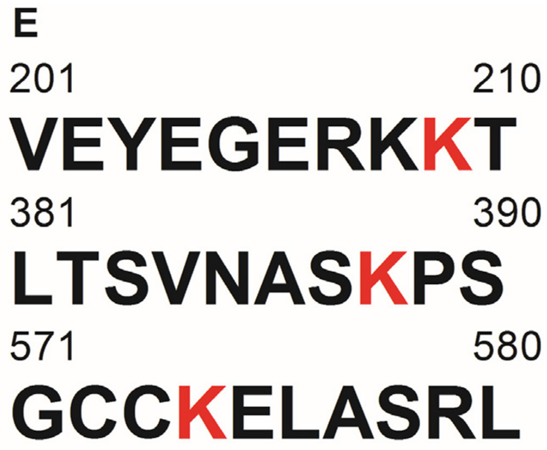

Supplementary Figure S2: (*Continued*) E. The localization the residues subject to modification (K209, K388 and K574). K in red indicates labeled lysine residues.

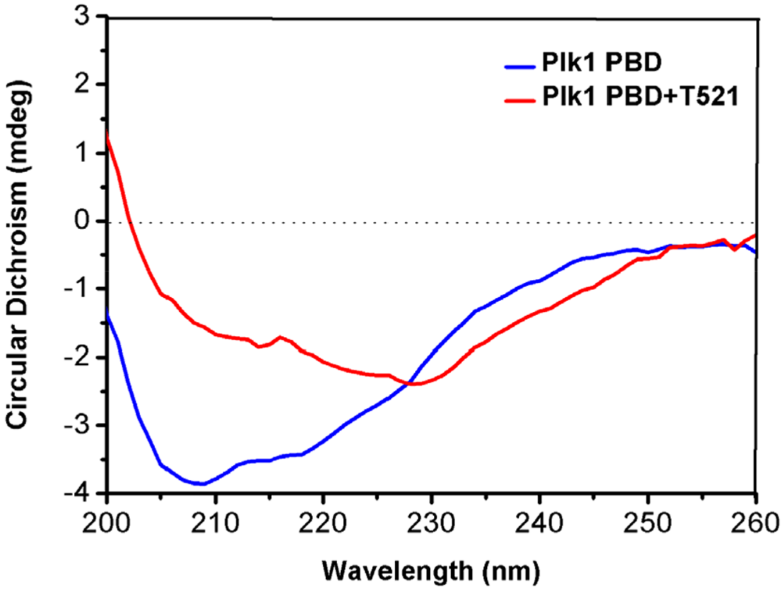

Supplementary Figure S3: The Far-UV CD spectra of Plk1 PBD. The CD spectra show the conformational change of Plk1 PBD in the presence of T521. PBS was used for all experiments.

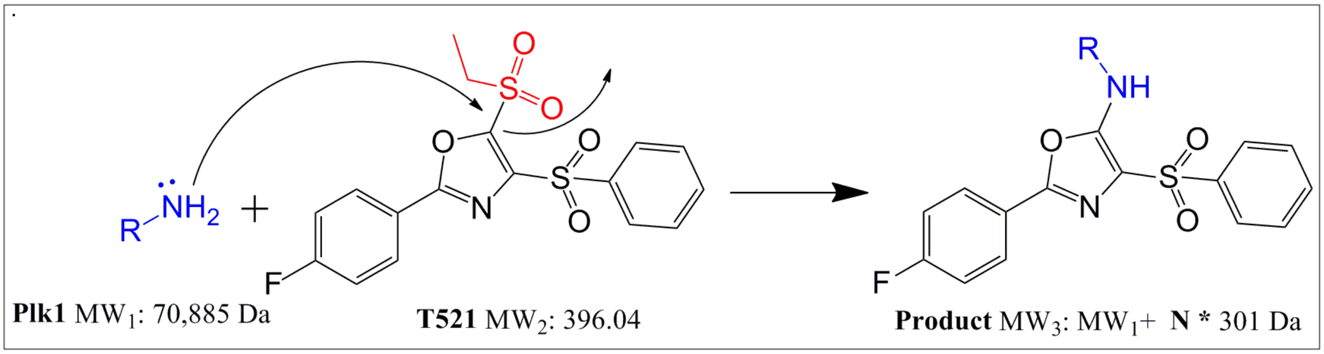

Supplementary Figure S4: The schematic illustration of covalent binding mode between T521 and Plk1. T521 is prone to a nucleophilic reaction because the carbon of oxazole linking ethylsulfonyl group in T521 shows more cationic (δ+), and is easy to be attacked by nucleophiles. The ethylsulfonyl group of T521 is a perfect leaving group, thus, T521 likely undergoes nucleophilic reaction. After attacking of T521 by the electron-rich alpha amino (alpha NH_2) of the K or R in Plk1, covalent addition of T521 to Plk1 protein could be triggered.
